# Open Reduction and Plate Fixation, External Fixator, and Conservative Treatment for Intra-articular Distal Radius Fractures

**DOI:** 10.7759/cureus.52014

**Published:** 2024-01-10

**Authors:** Mehmet Akdemir, Ali Īhsan Kiliç, Cengizhan Kurt, Sercan Çapkin

**Affiliations:** 1 Orthopedics, Izmir Ekol Hospital, Izmir, TUR; 2 Orthopedic Surgery, Izmir Bakircay University, Izmir, TUR; 3 Orthopedics and Traumatology, Izmir Bakircay University, Izmir, TUR; 4 Orthopedics and Traumatology, Faculty of Medicine, Aksaray Education and Research Hospital, Aksaray University, Aksaray, TUR

**Keywords:** intra-articular fracture, external fixation, volar plate fixation, conservative management, distal radius fractures

## Abstract

Background

Distal radius fractures are common fractures. Treatment of intra-articular fractures is controversial, with treatment modalities including closed reduction and casting, open reduction and plating, and closed reduction and fixation with an external fixator. In this study, we compared the clinical and radiological outcomes of our patients treated with three different methods for intra-articular distal radius fractures. We hypothesize that open reduction and plate application are superior.

Methodology

Adult patients with intra-articular (AO type B and C) fractures of the distal radius and treated with closed reduction-casting, volar locking plate, and external fixator were identified. Radiologically, joint stepping at the end of treatment, radial inclination, radial height, volar tilt, and distal ulna fracture were examined. For clinical scoring, Quick Disabilities of the Arm, Shoulder and Hand (Q-DASH) scores were computed. Obtained data were compared statistically between groups.

Results

A total of 164 patients were included in the study. Overall, the treatment modality was conservative in 61, volar with plating in 78, and external fixation in 25. The mean age of the patients was 53.7 years (range = 20-82 years). Overall, 39.6% of the patients were male and 60.4% were female. The mean follow-up period of the patients was 16.7 months (range = 12-28 months). No statistically significant difference was found between Q-DASH scores in the statistical evaluation (p > 0.05). There was a statistically significant difference between the groups in the radiological evaluation. When conservative treatment and the volar plate group were compared, the volar plate was superior in all radiological parameters (p < 0.05). Compared with conservative treatment and external fixation, only volar tilt and radial inclination angle were different. External fixation was better (p < 0.05). Radial length, volar compared to plate and external fixation tilt, and ulnar variance were better in the plate group (p < 0.05). Regarding joint stepping and radial, there was no difference in inclination between the two groups (p > 0.05). Reflex sympathetic dystrophy was seen in a total of 10 (6.1%) patients. Pin-site infection was seen in three (12%) patients in the external fixator group. Implant removal was performed in seven (9.0%) patients who developed plaque due to irritation and tenosynovitis. Early arthrosis was seen in three (4.3%) patients.

Conclusions

The treatment of distal radius intra-articular fractures should be evaluated and decided individually for each patient. No single method is directly superior to other methods. However, in some cases, the best results radiologically in the treatment of comminuted intra-articular fractures have been obtained in patients with volar plates.

## Introduction

Distal radius fractures are common fractures in all age groups. It can be seen in postmenopausal women and at a younger age in men due to simple falls and accidents [1]. Most fractures are extra-articular and can be treated conservatively [1,2]. However, the treatment of fractures extending to the joint with a large amount of depression is controversial. Cast after closed reduction, locked plate screw after open reduction, closed reduction, external fixation, and percutaneous pinning are the treatment options [1].

The treatment aims to enable the patient to regain their former functional level. In addition, it aims to minimize deformity and preserve the range of motion [1,2]. Therefore, open reduction and locking plate screws have become popular in recent years [3]. However, due to reasons such as soft tissue damage caused by open surgery, conservative treatment maintains its current position [2]. The most important problem in closed reduction is that the reduction cannot be achieved or preserved completely [4]. Another alternative method is the external fixator method, which can be applied both closed and the reduction can be better. The most important problem with this method is the difficulty in being tolerated by patients [5].

This study aims to investigate whether there is a clinical and radiological difference between the different treatment methods for intra-articular distal radius fractures. We hypothesize that the open reduction and plating method provides better results radiologically.

## Materials and methods

All patients were treated by a single orthopedic surgeon. Patients treated for distal radius fractures in our clinic were retrospectively scanned from the hospital system. Intra-articular fractures, reduction patients, and patients treated with the plate screw or external fixation method were identified. Patients with extra-articular fractures; patients followed conservatively with cast without reduction; patients under the age of 18; patients who did not undergo surgical treatment due to advanced comorbidity and were followed conservatively; patients with mental problems, pathological fractures, open fractures, bilateral fractures, and accompanying ipsilateral upper extremity fractures; and patients who underwent dorsal plating or volar non-locking plate were excluded from the study. The decision on which treatment to administer to a patient was based on both the surgeon’s and the patient's preference, but, most importantly, it was a joint decision made collaboratively.

Patients with initial fracture, post-reduction, and follow-up films were included in the study. Quick Disabilities of the Arm, Shoulder and Hand (Q-DASH) questionnaire was given to the patients for clinical scoring (minimum clinical follow-up period was 12 months). Fracture type on direct radiographs, joint stepping, radial inclination angle, radial height, volar tilt, and distal ulna fracture status were checked, and measurements were made via the PACS system. In addition, patients’ age, gender, side, and control time information were noted, and the data were entered into Microsoft Excel (Microsoft Corp., Redmond, WA, USA).

In all treatment modalities (closed reduction, volar plating, and external fixation), desired radiological values were <3-5 mm radial shortness, <10-15' dorsal angulation, <15' volar, <20' angulation, >15' radial inclination, and <1-2 mm segregation in intra-articular fragments [2,6].

**Figure 1:**
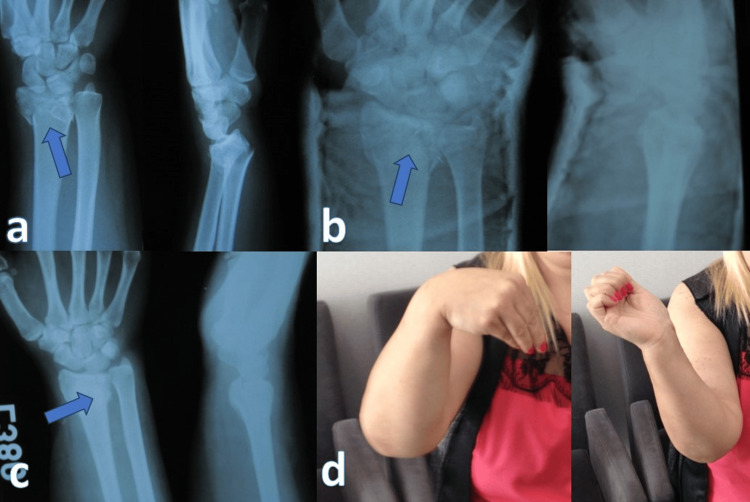
Conservative treatment. (a) Intra-articular comminuted distal radius fracture before reduction and intra-articular fragment (arrows). (b) Radiographic image of the distal radius in a cast after closed reduction. (c) Third-month control radiograph and first-year clinical image. (d) Good clinical outcome despite some dorsal angulation at the end of the follow-up.

The reduction of patients included in the study and treated with conservative treatment was performed under local anesthesia (hematoma block) or sedation (Figure 1). The reduction was done with traction. After the reduction, first, a short arm splint and then a circular short arm cast were replaced in the first week according to the control film. Finger and elbow movements were started immediately after reduction. It was followed with a cast for a month. After the plaster was removed, a control film was obtained. Subsequently, physical therapy was started with finger and wrist movements.

**Figure 2:**
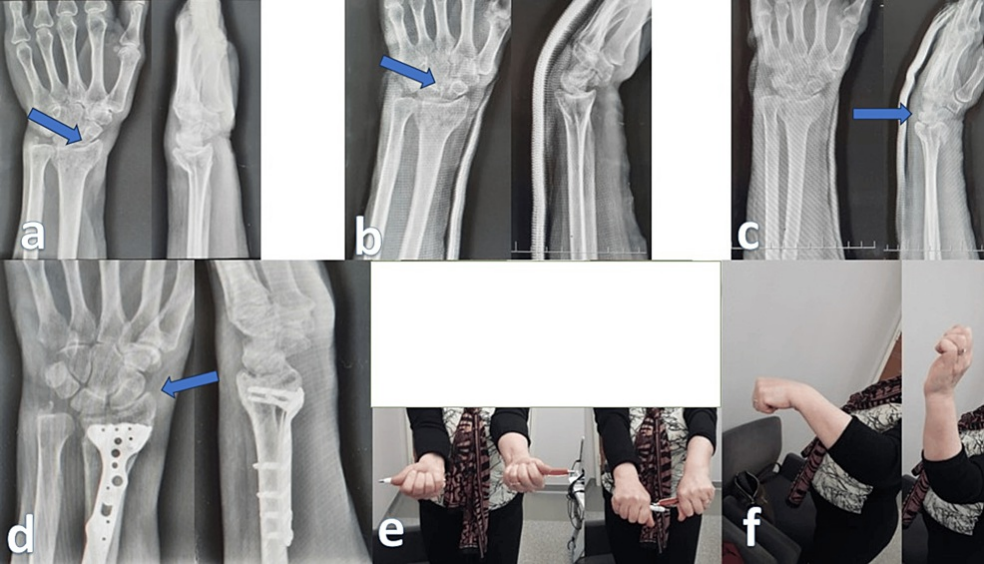
Volar plate. (a) Distal radius fracture before reduction with intra-articular fragmentation (arrows). (b) Closed reduction and casting treatment was tried on the patient in the first stage. (c) However, due to metaphyseal collapse and joint separation on the following radiographs (second and third weeks), (d) plate and screw fixation was performed on the patient. (e and f): Fourteenth-month control radiograph and clinical result.

Plate application was performed with a volar Henry incision under general anesthesia or axillary block. After fracture reduction, fixation was performed with a locked (fixed angle) plate-screw system. Finger movements were started immediately after surgery. During soft tissue healing (one to two weeks), it was followed with a short arm splint. Then, the splint was removed, and finger and wrist movements were started. One month later, radiography was checked (Figure 2).

**Figure 3:**
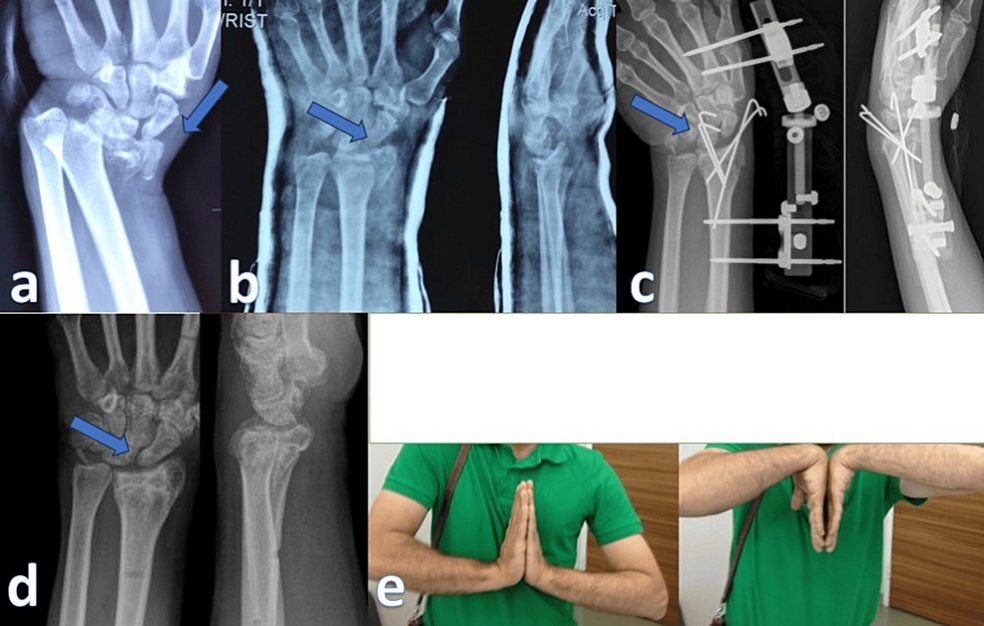
External fixation. (a) Intra-articular distal radius fracture due to high-energy injury before reduction. Arrows indicate the intra-articular fragment. (b) First, it was aimed to protect the neurovascular structures with closed reduction (control radiograph immediately after reduction). (c) However, due to joint stepping after reduction, the patient underwent joint reduction with external fixation and K-wires (three weeks after surgery). (d) Final radiograph at the 13th month (arrow indicates articular fragment). (e) Good clinical outcomes despite some metaphyseal collapse in the 13th month.

An external fixator was applied in the operating room under general anesthesia. After fracture reduction, joint restoration was performed with K wires if necessary. Fixation was done with two Schanz distally passing through the second metacarpal and two Schanz proximally to the radius with an articulated wrist external fixator (Figure 3). Traction was performed to provide minimal soft tissue tension. Finger movements were started immediately, and wrist movements were started after the soft tissue edema regressed (one to two weeks) by loosening the joint of the fixator. According to the first-month control, the fixator and K-wires were removed under local anesthesia in outpatient clinic conditions. A short arm static hand-wrist splint was used for one to two weeks after fixator removal. Active finger and wrist movements were continued.

To control for surgeon bias, radiological measurements and evaluations were done by two different authors. The consistency of two different measurements was evaluated statistically with the interclass correlation coefficient. Clinical scoring was done according to the Q-DASH scoring system. Q-DASH is a patient-oriented assessment. The Q-DASH form was given to the patients by the assistants working in the outpatient clinic and was filled in by the patients.

SPSS version 20 (IBM Corp., Armonk, NY, USA) was used for statistical evaluation. The chi-square test was used in the evaluation of cross-sectional data. Compliance with normal distribution in the evaluation of numerical and measurement values was determined by the Shapiro-Wilk test. The one-way analysis of variance (ANOVA) test was used in cases where the normal distribution was provided, and the Kruskal-Wallis test was used in cases where it did not fit. Statistical significance was accepted as p < 0.05. Post-hoc analysis of the values with significant differences in the ANOVA test was performed with the Games-Howell test, and in the Kruskal Wallis test, in groups of two, with the Mann-Whitney U test. Two different authors conducted the graphic measurements. The interclass correlation coefficient was used to perform a reliability analysis between the measurements. For an overall assessment, the average of the two different measurements was taken.

## Results

As many as 214 patients were identified. In the conservatively treated patient group, patients who were minimally displaced, treated with plaster without reduction, and who were operated on due to advanced age and additional comorbidities were eliminated. Finally, 164 patients who met the inclusion criteria were included in the study. Of the patients included in the study, 61 underwent conservative treatment (closed reduction and follow-up with cast), 25 were treated with closed reduction followed by a hand-wrist external fixator (auxiliary K-wire for the joint surface), and 78 were treated with a volar fixed angle (locked) plate treatment with a volar approach.

The mean age of the patients was 53.7 years (range = 20-82 years). Of the patients, 65 (39.6%) were male and 99 (60.4%) were female. The right side was operated in 90 (54.9%) patients, and the left side was operated in 74 (45.1%) patients. The mean follow-up period of the patients was 16.7 months (range = 12-28) months. All fractures were articular fractures. There were partial articular (AO type B) fractures in 74 (45.1%) patients and complete articular (AO type C) fractures in 90 (54.9%) patients. Distal ulna styloid fractures were present in 87 (53.0%) patients and absent in 77 (47.0%) patients. In the statistical evaluation, no statistically significant difference was found between age, gender, side, follow-up time, fracture type, and presence of distal ulna fracture (p < 0.050) (Tables 1, 2).

**Table 1:** General demographic data. *: chi-square test. F = female; M = male; R = right; L = left

		Conservative		Plate		External fixator		P-value
		N	%	N	%	N	%	
Number of patients		61	37.2	78	47.6	25	15.2	-
Gender	F	38	62.3	48	61.5	13	52.0	0.647*
	M	23	37.7	30	38.5	12	48.0	
Side	R	34	55.7	32	41.0	8	32.0	0.080*
	L	27	44.3	46	59.0	17	68.0	
Fracture type (AO)	B	30	49.2	31	39.7	13	45.1	0.408*
	C	31	50.8	47	60.3	12	54.9	
Distal ulna fracture	Yes	30	49.2	45	57.7	12	48.0	0.523*
	None	31	50.8	33	42.3	13	52.0	

**Table 2:** Clinical and radiological data. *: Kruskal-Wallis test; **: one-way analysis of variance SD = standard deviation; Q-DASH = Quick Disabilities of the Arm, Shoulder and Hand

	Conservative		Plate		External fixator		P-value
	Mean	SD	Mean	SD	Mean	SD	
Age	54.67	14.49	52.19	14.78	56.08	12.60	0.337*
Joint stepping (mm) (control)	0.516	0.60	0.282	0.46	0.32	0.53	0.031**
Radial length (mm)	12.26	3.87	14.25	3.99	12.52	3.12	0.006*
Volar tilt angle	-2.68	9.72	7.47	6.44	2.02	9.46	<0.001*
Radial inclination angle	19.66	4.91	23.49	3.75	24.06	4.01	<0.001*
Ulnar variance (mm)	-2.82	3.12	-0.76	1.72	-2.25	2.45	<0.001**
Follow-up (months)	16.07	3.49	17.15	4.06	16.52	4.11	0.256**
Q-DASH score	15.54	14.35	12.53	10.79	15.73	12.65	0.558*

No statistically significant difference was found between the three groups in the clinical scoring of Q-DASH (Kruskal-Wallis test; p = 0.558, p > 0.05) (Table 2). The reliability of the two measurements was assessed using the interclass correlation coefficient test. Cronbach’s alpha values were greater than 0.9 for all radiologic parameters. There was a statistically significant difference between the three groups in joint stepping, radial length, volar tilt angle, radial inclination angles, and amount of ulnar variance (ANOVA and Kruskall-Wallis test; p = 0.032, 0.005, <0.001, <0.001, <0.001, and <0.05) (Table 2). There was a significant difference in all parameters when the conservative and plate groups were compared using the Games-Howell post-hoc analysis and Mann-Whitney U test (Table 3). In the comparison of the conservative group and the external fixator, the radial inclination and volar, there was a significant difference in tilt angles, but no difference in other parameters (Table 3). On comparing plate and external fixator, volar, there was a significant difference in tilt angle, radial length, and amount of ulnar variance. There was no significant difference in the other groups (Table 3).

**Table 3:** Comparison of groups. *: Post-hoc analysis; Games-Howell; **: Mann-Whitney U test.

	Conservative - Plate		Conservative - External fixation		Plate - External fixation	
	P-value	Significance	P-value	Significance	P-value	Significance
Joint stepping	0.035*	Yes	0.296*	No	0.943*	No
Radial length	0.003**	Yes	0.749**	No	0.028**	Yes
Volar tilt	<0.001**	Yes	0.023**	Yes	0.009**	Yes
Ulnar variance	<0.001*	Yes	0.641*	No	0.021*	Yes
Radial inclination	<0.001**	Yes	0.001**	Yes	0.495**	No

Complex regional pain syndrome (CRPS) developed in 10 (6.1%) patients (six conservative, two plates, two external fixators). There was no statistically significant difference between the groups (Pearson chi-square test; p = 0.188, p > 0.05). Pin-site infection developed in three patients treated with external fixators (12.0%). Implant removal was performed due to pain and tenosynovitis in seven (9.0%) patients who underwent plate fixation. Early post-traumatic arthrosis developed in seven (4.3%) patients (two conservative, three external fixator, and two plate group patients). There was no statistically significant difference between the groups (Pearson chi-square test; p = 0.480, p > 0.05).

## Discussion

There is no agreement on the ideal treatment for distal radius fractures. The results of conservative treatment of minimally displaced, extra-articular fractures are satisfactory. However, the treatment of displaced fractures extending to the joint is controversial. Recommended treatment modalities are closed reduction and casting, external fixation and open reduction, and internal fixation. In recent studies, open reduction and treatment with volar anatomical plates and fixed-angle screws have come to the fore, especially in intra-articular fractures [7,8]. Studies have published good results, especially when external fixation is performed with the fixation of intra-articular fragments with K-wires [9].

Regarding conservative treatment and volar plate application, reduction quality was better in the volar plate group in almost all studies [7,8,10-12]. However, when the clinical results were compared, different results were found. While Saving et al. reported that the volar plate was superior in their study, Arora et al. could not find any difference between the two groups and even more complications in the plate-applied group [10,12]. In our study, we achieved better results in all radiological scores in the volar plate-applied group compared to the conservative group (Table 2). There was no significant difference between clinical Q-DASH scores. Studies have reported that values other than joint stepping in the radiological evaluation are not correlated with the clinical results, especially in elderly patients [13,14]. However, these values affect the cosmetic appearance of the patients. Although there is no clinical functional difference, this issue should be discussed with patients before treatment.

External fixation therapy, on the other hand, can be considered a suitable alternative method when combined with minimal invasiveness and fixation of fracture fragments with K-wire. In a study comparing external fixation and conservative treatment, better radiological results and wrist joint range of motion were obtained in patients who underwent external fixation, but similar results were obtained in functional scores (DASH) [9]. In our study, however, volar tilt and radial inclination angles were better in the external fixation group, while there was no significant difference in clinical scores. As there was no significant difference in joint stepping and clinical results in our study, we think that conservative treatment with external fixators led to very similar results.

Better grip strength and range of motion have been reported in patients with early volar plate application compared to those with external fixation [15,16]. In our study, clinical scores were similar between the volar plate group and the external fixator. Radiologically, we had better results in the volar plate group. An external fixator is a good alternative for patients with polytrauma, soft tissue defects, and closed reduction. However, it is not superior to conservative treatment in other patients.

The most common complication in our study was CRPS at a rate of 6.1% in 10 patients. This result was similar to the literature (4.6%) [17]. We performed implant removal due to tenosynovitis and pain in 7 (9%) patients in whom we applied a plate. This result was similar to the literature [17,18]. We did not encounter flexor or extensor rupture in patients who developed tenosynovitis, as we performed implant removal in the early period. We did not encounter the problem of screw loosening or penetration of the screw into the joint in our cases. We encountered early posttraumatic arthrosis in seven (4.3%) patients. We expect this rate to be lower than expected. This rate is likely to increase with longer follow-ups [19].

Limitations of our study are its retrospective design and the moderate number of patients. We do not routinely use CT for assessing patients’ fractures. We only employ it in selected cases. The use of CT both increases costs and results in additional radiation exposure. However, fracture assessment is better performed with CT. Although a one-year follow-up period is an appropriate follow-up period for distal radius fracture, a longer follow-up is required for the development of posttraumatic arthrosis. Intra-articular distal radius fractures represent a heterogeneous group. In addition, the comparison with three different treatment methods is a factor that makes the comparison difficult. For this reason, patients who were displaced and reduced, especially in the conservative patient group, were included in the study. Non-displaced and minimally displaced patients who were treated conservatively and did not need reduction were not included in the study. In addition, while patients with conservative follow-up are mostly older, surgical treatment is more preferred to young people. When our study was planned, patients over a certain age who were followed conservatively were not included in the study. Thus, it was aimed to make the groups more homogeneous.

## Conclusions

The treatment of distal radius intra-articular fractures should be evaluated and decided individually for each patient. No single method is superior to other methods. However, in some cases, the best results radiologically in the treatment of comminuted intra-articular fractures have been obtained in patients with volar plates. There is little radiological difference between the external fixator group and the conservative group. There was no significant difference between the three groups in the Q-DASH scores. Closed reduction should be attempted first in all intra-articular and comminuted distal radius fractures. In patients with appropriate alignment, treatment should primarily be conservative. In cases where proper alignment cannot be achieved or maintained, a volar plate should be preferred as the surgical fixation method. The use of an external fixator in comminuted intra-articular distal radius fractures is limited and has no or very little radiological and clinical advantage over conservative treatment.
